# Continuing Education in Irish Hospital Schools: Provision for and Challenges for Teachers

**DOI:** 10.5334/cie.25

**Published:** 2021-04-15

**Authors:** Sandra Keehan

**Affiliations:** 1 Children’s Ark School, IE

**Keywords:** Hospital schools, peripatetic teachers, paediatric hospital, hospitalised child

## Abstract

This qualitative research study examined the educational provision for children with medical needs in Irish hospital schools using a case study approach encompassing the perspectives and experiences of 12 teachers currently teaching in two paediatric hospital schools. Document analysis and semi-structured interviews were employed across two research sites, which included a review of each school’s whole-school evaluation, enrolment policy, school improvement plan, database, and school website, to compile information to inform each case study. Scrutiny of the documentation also assisted in the formation of the interview questions used in the semi-structured interviews. Thematic analysis was conducted on the data collated, and four key themes were identified.

The findings revealed that participating hospital teachers employ a variety of individual and collaborative practices to prepare for and deliver education to hospitalised students. The research illuminated the routine of planning for a child’s education, comprising a process of information gathering and recording, multi-disciplinary collaboration, and engagement strategies. Hospital teachers reported the emotional aspect of their role as the biggest challenge they experience, while time constraints, teaching across a variety of class levels and needs, and a lack of recognition of the parameters within they must operate were further challenges identified.

Continued medical developments have resulted in higher survival rates for those with complex and enduring medical conditions (Uggeri et al., 2016). With a greater number of at-risk children surviving, this in turn has led to an increase in the number of children who are required to manage a health condition during their critical school years (Eaton, 2012). Managing a health condition impacts every aspect of children’s development, including social relationships, self-esteem, academic performance and, ultimately, the ability to access the same educational outcomes as their healthy peers (Shiu, 2001). Shaw and McCabe (2008) note that the reduced class time associated with recurrent hospital visits results in decreased time spent learning core academic concepts and bonding with peers. The potentially damaging effect of such absences is further reiterated by Thies (1999), who notes that self-confidence, motivation, and achievement are undermined as the child falls behind academically, and proposes that when this occurs, efforts are directed towards catching up, which in turn takes time away from keeping up (p. 395). The negative impact of having to catch up on missed work, learning gaps due to lack of direct instruction, along with disrupted friendships, can individually and collectively create stress and anxiety (A’Bear, 2014).

Provision for education for these children during periods of hospitalisation is, therefore, a logical requirement to reduce the damaging effects caused by school absenteeism due to hospitalisation (Shaw & McCabe, 2008). Thus, hospital schools and their educators provide education to children during periods of hospitalisation to keep them up-to-date with their peers in mainstream schools (Uggeri et al., 2016).

St. Leger (2014) noted the dramatic effect that the behaviours of peers, teachers, parents, and other school staff can have on the psychological and academic outcomes of students with medical needs. Therefore, the response from a school setting can be influential on how a child engages with education in the long term. In a hospital school, hospital teachers strive to maintain normality for this cohort of learners while they are receiving treatment (Steinke et al., 2016), an educational intervention that Kaffenberger (2006) identified as an essential component towards supporting children whose illness and frequent hospitalisations have resulted in the impairment of their school participation.

While hospital schools seek to create a community of children and adolescents and normalise everyday life, the reality is that the setting is not a typical one, as hospital education is organised as class, group, or individual teaching conducted at the child’s bedside (Hospital Organisation of Pedagogues, Europe, [HOPE], 2000). To facilitate education, hospital teachers must provide instruction across many grade levels to children of varying need and ability, and engage in collaborative practice daily. This comprises an information-gathering process for each individual child to inform their educational process (Uggeri et al., 2015). Their role also necessitates a knowledge of the educational needs of the child as well as the characteristics of their medical treatment to formulate an appropriate plan for the child’s instruction, taking into account the likes and dislikes of the student, available learning time, and treatment side effects (Uggeri et al., 2015).

## Individual and Collaborative Practice

The necessity for teachers to work together to devise learning opportunities for pupils above and beyond the curriculum is identified in the framework compiled by the Department of Education and Skills ([DES], 2016), which outlines collaboration between teachers, parents, and relevant and appropriate outside personnel to provide meaningful learning experiences for pupils as effective practice. Alongside this, hospital teachers must be familiar with a variety of resources and grade-level curricula to be able to deliver education to whatever children present on any given day (Steinke et al., 2016). Similarly, HOPE (2000) holds as a principle that a variety of teaching methodologies and resources be utilised, the content of which should encompass more than formal curriculum learning, and include subjects related to special needs arising from illness and hospitalisation. To gain this requisite subject and pedagogical knowledge, engagement in Continuous Professional Development (CPD) is recognised as being intrinsic to effective collaborative teacher practice (DES, 2016; HOPE, 2000).

Supporting learners through their hospital education requires effective collaboration by maintaining contact with stakeholders involved in the child’s care, such as parents, medical staff, mainstream school, and peers (Uggeri et al., 2016). Emphasising the importance of such collaboration, Hopkins et al. (2014) maintain that learning in hospital works best when there are clear and open channels of communication between health professionals, educators, parents and, importantly, the child, to contribute to the child’s development, well-being, education, and socialisation. Collaborating with the student’s base school to obtain work and curricular information for the child is essential (Eaton, 2012), and fundamentally minimises educational losses (Borgioli & Kennedy, 2003). As many students are hospitalised with little or no prior warning and may be discharged without much prior notice, sustaining a long-term commitment to school curriculum objectives can prove problematic (Peters et al., 2016). It is therefore essential that hospital teachers remain committed to collaborating with stakeholders involved in the child’s education to ensure lines of communication are kept clear and open. Through organisation of educational programmes designed to follow the objectives and approaches of the learner’s mainstream school, preparatory work can be undertaken by teachers for the child’s eventual or potential return to mainstream school upon discharge (Uggeri et al., 2016).

Disease itself or its medical treatment may lead to a small but significant group of students experiencing an obvious drop in performance (Shiu, 2001). To address this, Shiu (2001) advocates the use of individualised educational programmes to assist students in fully accessing the curriculum. The unique contextual elements of hospital teaching also necessitates that hospital educators demonstrate a considerable capacity for flexibility in the organisation of their work and in their instructional planning (Benigno & Fante, 2020). Planning clear and relevant learning objectives that are contextualised to pupils’ learning needs, as well as preparing for differentiation of learning objectives and activities, including personalised learning opportunities, are hallmarks of teachers’ effective practice as outlined by DES (2016), and require strategic selection and use of approaches to match the learning objective of the lesson and meet the learning needs of the pupils. In sum, delivery of instruction should elicit pupil engagement and ensure that meaningfully differentiated content ensures that all students experience success as learners (DES, 2016).

## The Importance of Communication

Wadley and colleagues (2014) noted the negative influence that school absences and disconnection from teachers and peers can have on a child’s attitude towards school, while findings from Crump et al. (2013) show that children living with chronic health conditions are at higher risk of disengaging prematurely from education than their healthy peers. Lost opportunities for the development of social relationships may increase social isolation in students with medical conditions as they experience a lack of connectedness to their peers (Kirkpatrick, 2020).

Ensuring that children remain connected to their school and keeping them interested and engaged in their education is imperative in order to avoid such negative attitudes and lack of motivation. This requires, in turn, that the school, classroom teacher, and the child’s classmates keep the absent child in mind as both a friend and a learner (Yates et al., 2010). The role of the hospital teacher was recognised by Eaton (2012) as beneficial to the child in achieving success and being the bridge that connects students to their peers.

To maintain connections with peers and base schools during periods of school absence, many teachers and researchers advocate the use of Information and Communication Technology (ICT) as a resource (Cashin & Witt, 2010; HOPE, 2000; St. Leger, 2014; Wadley et al., 2014). When appropriately designed and respectful of sensitive context, ICT can help improve the emotional well-being of children who are hospitalised and can reduce isolation (Wadley et al., 2014). Teachers who facilitate ICT communication recognise the connection to school as critical in terms of the child’s social network, sense of worth, and identity (Dixon, 2014).

The importance of liaising with the base school of a child with medical needs has been explored in the literature (Capurso & Dennis 2017; Steinke et al., 2016; Wadley et al., 2014). Its benefits include the creation of more personalised educational experiences that take into account personal learning styles and prior experiences as well as the possibility of obtaining a comprehensive educational programme to follow during periods of hospitalisation.

An essential component of the effectiveness of hospital teachers is collaboration with medical and psychosocial staff (Benigno & Fante, 2020; Steinke et al., 2016). Enabling teachers to understand the young person’s particular health condition and how it may impair him or her in accessing education requires effective communication in this area (Hopkins et al., 2014). Furthermore, it is necessary to recognise parents as active and responsible partners who must be informed about the right to education and the programme of education for their child (HOPE, 2000).

Bidirectional collaboration between parents, teachers, and medical staff is reflective of effective practice. While teachers require relevant information about the child’s condition and capabilities, medical staff and parents require relevant information relating to the pedagogical approach being undertaken and the efforts required to provide such support (Uggeri et al., 2016). Home-school collaboration and partnership is essential to promote successful school work at hospital schools, and the special expertise required to build such relationships is a characteristic of hospital pedagogy (Äärelä et al., 2018).

## Challenges Experienced by Hospital Teachers

The prospect of facing the death of a student is an unfortunate reality of working with children with medical needs, and has been cited in literature as a significant challenge for hospital teachers (Lemke, 2004; Steinke et al., 2016). To deal with this unique variable, it is essential that hospital school programmes plan for staff support surrounding grief and bereavement, while leaders must promote self-care to ensure that staff members are equipped to adequately cope in such an event (Lemke, 2004).

Further challenges faced by hospital teachers include time constraints, hospital politics, lack of recognition and support, and lack of appropriate educational resources (Steinke et al., 2016) as well as fluctuations in enrolment and difficulties connected to trying to engage students during short admissions (Lemke, 2004). Steinke et al. (2016) emphasised that a closer look at the demands on a hospital teacher’s time is necessary to determine appropriate student-teacher ratios and other staffing demands for hospital schools. Unlike a typical school setting where children are presented to a teacher daily, in a hospital setting, teachers spend a significant amount of time canvassing for potential students. This can prove a challenge due to time constraints, as it involves reviewing the hospital admissions list for school-age patients, rescheduling pre-planned school sessions due to procedures or severity of illness, and communicating with the child, parents, and medical team in the hospital (Steinke et al., 2016).

## The Present Study

In Ireland, hospital schools are not categorised separately from mainstream and special schools in the Key Statistics National School Census as issued by the Department of Education and Skills ([DES], 2018). The researcher, through prior knowledge of the hospital school system in Ireland and through consultation with colleagues, identified 12 schools catering specifically for children with medical needs but acknowledges that more may exist that are not specifically referred to as “hospital schools” in the census. Among these schools are schools catering for children who attend the country’s acute tertiary national paediatric hospitals, children who attend for services on paediatric wards in a number of hospitals, children who attend Child and Adolescent Mental Health Service (CAMHS) centres, and children who attend rehabilitation services.

Existing research reports that provision for education for children in hospital is fundamentally beneficial for students with medical needs; however, a review of the literature suggests that there is little to no research on this area of education from an Irish perspective. To explore where Ireland lies within the context of provision for education for students with medical needs, particularly the practices they employ and the challenges they face, the following research questions were identified to be investigated in the current study:

1. What examples of teachers’ practice, both individual and collaborative, are evident in Irish hospital schools?
2. What are the challenges experienced by teachers in Irish hospital schools?

## Method

### Design

A qualitative research design using an inductive approach was chosen in order to examine the educational provision for children with medical needs in Irish hospital schools and to capture the complexity of people’s meanings and experiences in the area of hospital education. A case study approach was adopted encompassing the perspectives of hospital teachers teaching in two paediatric hospital schools to identify the individual and collaborative practices employed (Yin, 2014). The schools involved in the case study were strategically selected based on their size to enable access to the greatest number of staff and data.

Semi-structured interviews and document analysis were employed across the two schools involved in the case study, from which the data were analysed to provide an insight into hospital school provision in Ireland. Document analysis involved a review of each school’s whole-school evaluation, school improvement plan, enrolment policy, database, and school websites. Semi-structured interviews were subsequently conducted with staff in the two participating schools (Supplementary File 1: Appendix A. Interview schedule). Thematic analysis was conducted on the data collated, and four key themes were generated (Braun & Clarke, 2006).

### Participants

Request letters to conduct research were sent to the principals of the respective schools detailing the purpose of the research, the intended method of data collection, and the time span of the data retention. Prior to any attempt at data collection, access to the hospital school and its staff was approved and written consent was obtained from the respective principals. Participants were invited to participate in the research upon meeting the criteria of being a qualified teacher currently employed full-time in the hospital schools involved. No part-time staff were employed at either of the hospital schools at the time of research. All teachers invited to be part of the study agreed to participate. These consisted of eight primary teachers and four post-primary teachers.

Signed consent was obtained from each participant prior to data collection. Confidentiality and anonymity was ensured for both schools and all participants and was presented both in writing and as a verbal reminder prior to commencement of the study. Pseudonyms were used for the school names and addresses. Further, participants’ identities were anonymised by assigning pseudonyms in the transcripts and removing any identifiable information from each transcript where relevant. By ensuring neither schools nor participants were identifiable, responses were not attributed to individuals. The anonymised participants are presented in ***Table 1*** below.

**Table 1:** List of Participants.


TEACHER	PRIMARY/POST-PRIMARY	YEARS OF TEACHING EXPERIENCE (HOSPITAL)	YEARS OF TEACHING EXPERIENCE (OTHER)	SCHOOL

Teacher 1	Primary	8.5 years	4 months	A

Teacher 2	Primary	11 months	12 years	A

Teacher 3	Primary	14 years	11 years	A

Teacher 4	Primary	19 years	20 years	A

Teacher 5	Post-Primary	15 years	1 year	A

Teacher 6	Post-Primary	6 months	17 years	A

Teacher 7	Post-Primary	3 years	8 years	A

Teacher 8	Primary	22 years	1 year	B

Teacher 9	Primary	8 years	4 years	B

Teacher 10	Primary	3 years	3 years	B

Teacher 11	Primary	20+ years	10+ years	B

Teacher 12	Post-Primary	5 years	20+ years	B


### Data Analysis

Thematic analysis was used to identify, analyse, and interpret the themes that were generated from the qualitative data retrieved (Braun & Clarke, 2017). Semi-structured interviews were conducted by the researcher, a fully qualified primary teacher, teaching in one of the schools involved in the research study, and were transcribed verbatim. The researcher, guided by a supervisor, conducted coding independently by generating initial codes from the discussion points collected during interviews, collating and analysing content to produce subthemes (Braun & Clarke, 2006). The identified subthemes were reviewed and refined to form distinct themes to capture the essence of the data (Braun & Clarke, 2006) (see ***Table 2***).

**Table 2:** Themes and Subthemes.


THEME	SUBTHEME

**Teacher attitudes towards supporting children in hospital**	**Continuing Education** Bridging gaps Reducing disengagement

	**Providing Normality** Communication with base school

**Routine of planning for a child’s education**	**Organisational Routine** Database Long-term planning Short-term planning Differentiation

	**Relational Dimension of Communication** Liaison with hospital staff Liaison with colleagues Engaging students

**Sharing knowledge**	***Teachmeet***

	**International Collaboration**

	**Medical Education Sessions**

	**Continuous Professional Development**

**Challenges experienced**	**Challenges Experienced** Bereavement Time constraints Bureaucracy

	**Supports** Support of colleagues PDST SESS


The themes generated through thematic analysis were as follows: (a) *Teacher Attitudes Towards Supporting Children in Hospital*, (b) *Routine of Planning for a Child’s Education*, (c) *Sharing Knowledge*, and (d) *Challenges Experienced*. These themes provided a framework for organising and reporting qualitative data pertaining to the research questions (Braun & Clarke, 2013), and give examples of teachers’ practice, both individual and collaborative, as well as the challenges they experience.

Adopting the approach of Hill et al. (2005), when determining the response of the 12 participants in the process of data analysis, a citation was termed as “most participants/there was consensus” when cited by more than 50% of participants. Themes cited by fewer than 50% of participants but not less than 25% were identified as “some participants”, whereas themes referred to by 100% of those interviewed were reported as “all participants”.

## Results

Document analysis concluded that both schools are situated in acute paediatric hospitals that act as national centres for a range of specialist medical conditions. The pupils of both hospital schools present with a wide range of learning needs to which the staff must respond with flexibility and creativity while providing for education in a range of settings, including the school’s main classroom, satellite classrooms on the ward, at the bedside on the pupils’ wards, and in isolation units.

At the primary level, hospital teachers teach the primary school curriculum as published by the National Council for Curriculum and Assessment ([NCCA], 1999), incorporating Aistear, the early childhood curriculum framework for early years (NCCA, 2009a), and also employ the guidelines for teachers of students with general learning disabilities (NCCA, 2007), where appropriate. At the post-primary level, the framework for junior cycle (NCCA, 2015) and senior cycle key skills framework (NCCA, 2009b) are employed. Both schools are recognised exam centres allowing pupils to sit their state exams when appropriate.

**School A** is responsible for the provision of quaternary, tertiary, and secondary healthcare services for children. The hospital is the national centre for a range of specialist areas, including children’s childhood cancers and blood disorders, cardiac disease, major burns, orthopaedics, cystic fibrosis, clinical genetics, and rheumatology, among other specialties.

Comprising one main classroom and one satellite classroom situated on a ward, the school has been regulated and funded by the Department of Education since 1964. Student enrolment numbers average at 80 daily, necessitating a daily pupil-teacher ratio of 10:1. School A’s vision has three objectives: (a) to provide a high-quality education service—appropriate, holistic and customised; (b) to maximise learning opportunities for students whose attendance has been compromised; and (c) to empower students to leave hospital with a positive attitude towards continuing their education.

**School B** is located in an acute paediatric hospital. Specialities in this setting include paediatric surgery, neurosurgery, nephrology, including haemodialysis, cystic fibrosis, metabolic disorders, craniofacial disorders, among others. Funded by the Department of Education since 1975, students of this school have access to one main classroom and two satellite classrooms located on two separate wards. The pupil-teacher ratio is 10:1, with an average daily enrolment of 50. School B’s mission statement recognises school as an integral part of the hospital, which delivers a holistic approach to the children, their parents, families, and staff. With an emphasis on continuing the academic education of each child, School B strives to build the child’s self-esteem, social and spiritual awareness, and prides itself on being a centre where love, freedom, justice, compassion, and joy find expression.

The four key themes generated from thematic analysis (*Teacher Attitudes Towards Supporting Children in Hospital, Routine of Planning for a Child’s Education, Sharing Knowledge*, and *Challenges Experienced*) represent the information required to provide a greater understanding of the service Irish hospital schools provide for students with medical needs and the practices undertaken to do so.

### Theme One: Teacher Attitudes Towards Supporting Children in Hospital

There was consensus among the participants that continuing education in hospital school relies on relationships, the relationship with the child being the most important. Teachers stressed the importance of continuing education and providing normality for successfully delivering education to support children in hospital schools.

#### Continuing Education

Participants corroborated the benefits and importance of continuing education across the data set. Thus, bridging the gaps and continuing the academic education for the hospitalised students was reported as essential by all participants. The necessity of continuity of education and maintaining a connection to school was acknowledged as an essential element of hospital teachers’ role as they strive to reduce disengagement from school and enable the children to return to their own schools post treatment. In the words of one of the participants: “I think it is a big stepping stone to getting back to school and it makes that daunting task of facing the teacher and the class when they go back a bit easier” (Teacher 8).

The importance of engagement not necessarily from an academic perspective was remarked upon by some participants as essential practice to keep children engaged in education, with one interviewee expressing the following view:

I suppose what we represent, is that education is important no matter what they’re going through in life at the time, and it might not be your straight academic work but that kind of connection to school I think is very important to them (Teacher 5)

Similar attitudes arose from the data regarding the importance of continuing education within a hospital setting to promote motivation and reduce disengagement; “Their motivation just weakens if they don’t attend school, it’s just like if you don’t exercise, you’re less motivated to go back and do it again” (Teacher 6).

The psychological benefit of education, essential in instilling in the students the message of hope in their own future, was expressed as an attitude of one participant, who noted that prior to illness, education is often regarded as of paramount importance to many families. Removal of the emphasis on the importance of education upon diagnosis “causes to increase the perception of how serious things are for the child” (Teacher 5).

#### Providing Normality

All teachers reported the uniqueness of the hospital school as a teaching environment. There was consensus regarding the facilitation of normality for a student as an essential component of a hospital teacher’s role. Teachers described the practice they engage in as they strive to facilitate normality within this unique environment by providing students with school that they are familiar with. Staff are committed to creating a setting that allows the children to feel as at home as possible. Interviewees described the benefits of providing the normality of school, remarking that it reduces the risk of student disengagement as they strive to reduce levels of anxiety in children.

Most teachers reported maintaining contact with the child’s class and class teacher as essential in providing normality for students. Teachers noted the use of Skype on occasion and writing letters with the students to send directly to their classmates as a means of maintaining connections.

### Theme Two: Routine of Planning for a Child’s Education

A significant finding that emerged from the analysis of the data set of both schools is that of the similarity in the routines of the participating hospital schools.

#### Organisational Routine

##### Database

All participants reported utilising each school’s database system as their method of officially recording information. Staff acknowledged the usefulness of having their database system directly linked with the main hospital admissions list to identify children of school-going age who are in-patients in the hospital.

Perusal of the database revealed that the system has the capacity to record a variety of information relating to each particular child. Personal information is recorded, to include date of birth, parent or guardian’s contact details, and isolation status. From an educational standpoint, the child’s class level, school contact details, and Individual Educational Plan (IEP) may be recorded, as well as any communications with base schools and information relating to the child’s education while in hospital.

The database serves as the school’s official roll, and reports of the record of work covered in hospital school as well as notes and observations on the students who attend are generated and circulated to the child’s base school and parents upon request. For General Data Protection Regulation (GDPR) adherence and patient confidentiality, the system is password-protected and only accessible on each school’s internal internet server.

##### Planning for a Long-Term Student

Teachers in both schools corroborated a similar means of planning for a child’s education during hospitalisation. Reporting that all curricular areas are covered in hospital school, teachers explained that yearly plans in each curricular area are developed at the beginning of each school year.

Consent is obtained from the child’s parents/guardians to contact a child’s base school in the event of a long hospitalisation or for a recurring student. Such contact involves hospital teachers liaising with base school teachers and grade-level heads to formulate an appropriate educational plan for the duration of the child’s hospital stay. Most participants noted that this communication is essential in determining a child’s base line and what to focus on, continue with, or revise. Where necessary, IEPs and plans of work are shared to assist the hospital teachers’ continuation of education with the child. One teacher (Teacher 9) remarked that this communication is mutually beneficial and equally important for the hospital teacher to be in the position to communicate what base line a child may be returning to his mainstream school with.

##### Short-Term Planning

Participants agreed that short-term planning is more generic in nature. All participants described using what they termed “newsletters” in their short-term planning. Described as theme-based resources created by the teachers to engage, assess, and differentiate content for each learner, newsletters comprise a four-page printed booklet with high-interest activities based on a specific theme for the child to complete. Teachers create newsletters under agreed-upon themes for each class group, incorporating the prioritised subjects of social, personal and health education (SPHE), numeracy, and literacy. Teachers base the themes of newsletters on what Irish schools may be covering at a particular time of year but are not tailored to specific students. All teachers reported that newsletters serve as effective tools to engage children while assessing what class level students are operating at.

### Relational Dimension of Communication

#### Liaison with Hospital Staff

All teachers described a morning routine of attending their assigned wards at the beginning of each school day. A summary of each child of school-going age on the ward is sought from the ward’s Clinical Nurse Manager (CNM). All teachers acknowledged the necessity of this liaison to inform of the current status of each child, whether they may receive bedside teaching, classroom teaching or whether they are too ill to receive an educational session. Upon clearance from the ward’s CNM, all participants referred to the routine of liaising with hospital school colleagues to share pertinent information relating to the students.

#### Engaging Students

All teachers described the strategies they utilise for engaging with students in hospital school. Interviewees described the process of relationship building as being conducted in a gentle manner, relying upon conversations with the children themselves and their parents to learn about the interests and dislikes of the child to assist in establishing a relationship.

All teachers detailed collaboration with stakeholders involved in the child’s care such as parents, base schools, and medical professionals as a method of gathering information, which all teachers deemed an essential practice in preparing an appropriately designed educational programme for a child and promoting engagement. All teachers referred to the essential practice of facilitating opportunities for socialisation between children as a priority and something to be promoted and encouraged whenever possible given the medical status of the child.

Most interviewees reported using the child’s own interests to initiate school sessions, especially on the first meeting with the child. High-interest individualised activities, with an emphasis on engagement and fun, are used to appeal to each individual student and promote effective relationship building. “We try to maximise learning opportunities for pupils as much as we can by offering high-interest lessons” (Teacher 7).

Some teachers observed that initially the emphasis may not be on academics but more on engagement with a social aspect and mindful of their medical status. One teacher reported the importance of engaging each child gently with his or her medical condition in mind: “Children who aren’t ready to physically engage in school, maybe it might be to sit at the bedside and read them a story … you have to take a different approach depending on the medical need” (Teacher 9).

Most participants acknowledged that while they encounter reluctant learners, for the most part this is not a frequent occurrence. On the occasion when they do meet reluctant learners, one participant remarked on the importance of persisting with offering an education service daily, with the overall experience being that such students are usually won over with persistence (Teacher 5).

### Theme Three: Sharing Knowledge

Interviewees reported many instances of engaging in collaborative practices as part of their work. Sharing information and knowledge was a recurring theme across the data set.

#### Teachmeet

Most teachers across the data set identified *Teachmeet* as an effective strategy by which to share teaching methodologies among hospital teachers. Specifically, they described *Teachmeet* as an opportunity for hospital teachers to promote professional collaboration and share an element of best practice. To facilitate this collaboration, three Irish hospital schools come together several times during the year, with speakers presenting for 7 minutes on a resource, activity, or methodology for hospital teaching that they find useful (Teacher 12). This sharing of knowledge has anecdotally been beneficial to hospital teachers to learn of effective strategies that they might like to try with their own students.

#### International Collaboration

The research found that all participants are members of the Hospital Organisation of Pedagogues, Europe (HOPE). Many acknowledged the benefits of attending biennial HOPE conferences to gather other perspectives and learn about resources used in other hospital schools, as well as having the opportunity to present hospital teaching practices from an Irish perspective. Some participants reported that sharing practice with other teachers in similar settings reduces the isolation of the hospital teacher that is felt on occasion. Two respondents advocated for participation in European Union-funded educational projects such as Comenius and Erasmus as an opportunity to engage in learning experiences targeted solely to hospital teachers.

#### Medical Education Sessions

Most participants reported noticing a variety of side effects of specific medical conditions such as fatigue, nausea, pain, low energy levels, and poor concentration levels in their students. One teacher remarked, “Chemo can affect a child’s ability to learn, it can affect their eyesight, their hearing. It will, if not compensated for, affect their ability to learn” (Teacher 4), while another noted that “All of our students have temporarily a special educational need due to their medical condition” (Teacher 5).

To address this need for information relating to medical conditions, annual education sessions delivered by medical professionals were cited by the majority of participants as being of great importance to their practice. Medical education sessions are facilitated by specialists in various disciplines (e.g., cardiac, burns, epilepsy, cystic fibrosis) to the whole staff to enable them to become better informed on the effects of medical conditions. Information relating to the medical condition itself and the effect that an illness may have on a child’s propensity to access education is imparted. Most teachers found this to be of immense importance as they require knowledge of what to look out for in a particular medical condition and how best to tailor educational programmes to cater for the challenges posed by different medical conditions.

#### Multidisciplinary Meetings (MDT)

According to interviewees, weekly attendance at psychosocial meetings is undertaken as part of their practice. Participants indicated that information exchanged at psychosocial meetings is beneficial as, through discussion of specific pupil cases, the hospital school team are informed of relevant information to inform their process (e.g., children with a new medical diagnosis, children transitioning to palliative care, and children who may have passed away). Teachers reported this knowledge as being of paramount importance when engaging with children and their parents. Academic-related content was also reported as a topic of discussion at such meetings on occasion. Teachers noted that MDT meetings may also advise on what the child may be able for academically, which in turn may be written into their educational plan.

Inclusion on the MDT was cited by one teacher as being beneficial not solely for the teacher but also for representatives of the other disciplines as information is shared from the school staff back to the team. “It [school] stimulates their brain again, and school is an essential therapy as such because we can pinpoint significant deficits … that might be occurring post event” (Teacher 9). Such information was cited as being important for the medical team to be made aware of.

#### Continuous Professional Development (CPD)

All teachers emphasised the importance of engaging in annual CPD to keep abreast with the range of educational developments that are utilised around the country in order to deliver appropriate and relevant education to the wide range of children they meet who come from a variety of educational settings. “You’re looking at the wide spectrum so you have to know what’s out there and what’s available” (Teacher 11).

### Theme Four: Challenges Experienced

Findings from participants’ perspectives across the data set reflect an overwhelming consensus that the emotional aspect of working as a hospital teacher is the biggest challenge faced when working in a hospital setting. Nine teachers cited children passing away as their most difficult challenge, while learning that a child has transitioned to palliative care was cited by two teachers as a further challenge. Some participants recognised that hospital teachers are very involved with the parents and families as well as the child themselves in their daily practice, an involvement that compounds the effect of bereavement and the challenge it poses for the staff.

Teaching across a variety of class levels was found to be a further challenge experienced by hospital teachers. Specifically, the differentiation required to effectively provide for education and, consequently, the requirement of having knowledge of and being familiar with the content for every class level. Furthermore, teachers must be familiar with the expectations of the delivery of a spiral curriculum, that is, revisiting educational topics over the course of a student’s education, with each encounter increasing in complexity and reinforcing previous learning.

Three teachers identified the uncertainty of each day regarding prioritising patients and being flexible with time as a difficulty encountered in the daily practice of hospital education. Some teachers reported not having time to teach all available children on their assigned ward as well as education sessions being too short, as a difficulty faced.

One teacher cited bureaucracy as a main challenge of hospital teaching, citing the challenge of having to fit within the parameters given to mainstream schools during Department of Education inspections, such as whole-school evaluations, as frustrating.

#### Supports

With regard to the support that is in place to assist hospital teachers in dealing with the challenges they face, one of the main supports was that of colleagues. While debriefing sessions and supervision as a staff to discuss concerns are available to staff, the majority of participants reported relationships and conversations with their immediate colleagues to be their greatest support.

With regard to supporting the variety of children teachers are required to teach, some teachers cited the Special Education Support Service (SESS) and Professional Development Service for Teachers (PDST) as valuable resources for providing CPD and inservice specifically tailored to the hospital setting.

## Discussion

The findings of the present study provide strong evidence to suggest that Irish hospital schools operate in a manner similar to the international hospital schools as discussed in the literature. Document analysis found that Irish hospital schools aim to continue education for children within the hospital environment by providing educational interventions to reduce the potentially damaging effect of a reduction in time spent learning core academic concepts and bonding with peers to which school absence is a contributor (Kaffenberger, 2006; Shaw & McCabe, 2008). Further, findings detailing common work practices that Irish hospital teachers engage in correlate with Uggeri et al. (2015), who reported that instruction is provided across many class levels with varying need and ability.

### Teacher Attitudes Towards Supporting Children in Hospital

The psychological benefits of continuing education were expressed by study participants, with self-esteem and motivation cited as areas at risk, corroborating findings by St. Leger (2014), Uggeri et al. (2016), and West et al. (2013).

Provision of opportunities for socialisation, applying an educational structure, and continuing academic development were reported as essential features of the role as a hospital teacher in Ireland (Eaton, 2012; Kirkpatrick, 2020; Lemke, 2004; Steinke et al., 2016). Efforts to maintain connections between students and their peers to develop and maintain healthy peer relationships are in evidence in Irish hospital schools and are consistent with international practice (De Rosier et al., 1994; Shiu, 2001; Wadley et al., 2014; Yates et al., 2010). ICT is utilised to support students to stay connected with their peers, a finding that is validated by research conducted by (Cashin & Witt, 2010; St. Leger, 2014).

### Routine of Planning for a Child’s Education

Two major areas under this theme were organisational routines and the relational dimension of communication.

#### Organisational Routine

Participants systematically record information on their school’s database system following oral communications with relevant stakeholders to populate the database with uniform information for each student. The use of comprehensive formal questionnaires to gather data (Uggeri et al., 2016) was not found to be a practice undertaken in this case study, as participants considered the methods currently in place as being more than adequate to collate necessary information.

Consistent with Wadley et al. (2014) and best practice guidelines as advised by the American Academy of Pediatrics ([AAP], 2000), this study found that teachers in Irish hospital schools strive to promote and sustain meaningful relationships between the child and their base school and endeavour to follow the child’s base school plan of work, when appropriate, to inform the educational programme for specific children. This practice echoes Capurso and Dennis’ (2017) emphasis on the benefits of acquiring requisite information relating to the child’s learning style or experience for the development of a more personalised educational experience, and is further advocated by Borgioli and Kennedy (2003) and Eaton (2012) as a recommendation of effective practice.

Peters et al. (2016) discussed the benefits of the implementation of Individual Learning Plans (ILPs) in patients’ medical records as a means of adopting a more personalised learning model for every priority patient. However, the current study found that hospital schools in Ireland do not currently include their education plans as part of the child’s medical record, instead opting to share assessments and reports with the child, parents, base schools, and the medical team, where appropriate and on request. Such bidirectional collaboration is in accordance with Uggeri et al.’s (2016) argument for its benefits in catering for the holistic development of the child.

All teachers reported the use of what they termed *newsletters* to support their short-term planning. This resource, described as a personalised method of reaching out to children of varying ages and ability while allowing for differentiation, engagement, and assessment, was found to be unique to Irish hospital schools as similar methodologies were not reported in the literature.

#### Relational Dimension of Communication

All teachers reported building relationships as essential to engaging students. Furthermore, all participants noted the necessity of gathering information through a collaborative approach to assist in formulating an appropriate educational plan, a practice that correlates with findings from Uggeri et al. (2015). DES (2016) advocates collaboration between relevant and appropriate outside personnel to provide meaningful learning experiences for pupils.

Participants in the present study affirmed that this effective practice takes place in hospital schools, with interviewees concurring that it is an essential part of their role in creating an educational programme. Specifically, most hospital teachers reported using high-interest, individualised activities to build relationships and encourage school engagement in hospitalised students. This finding correlates with the DES (2016) framework’s proposal of using personalised learning opportunities that elicit pupil engagement by ensuring that meaningful and differentiated content and activities allow for all students to experience success as learners.

The data suggest that hospital teachers in Ireland demonstrate an understanding of the importance of considering medical needs when engaging students in educational sessions. The data further revealed that teachers demonstrate a knowledge of when to curtail work, engage in proactive gentle engagement strategies, and adapt the physical learning environment to provide familiarity, as advocated by Eaton (2012) as essential to a hospital teacher’s role.

### Sharing Knowledge

Liaison with medical staff is considered an essential component for hospital teachers to be effective (HOPE, 2000; Steinke et al., 2016; Uggeri et al., 2016). Findings from this research study report that all teachers liaise daily with clinical nurse managers, nursing staff, health care assistants, and other medical professionals to gather information relating to the condition of the children on the wards and to share advice relating to implementing an appropriate educational programme on any given day.

This practice correlates with best practice as advised by Steinke et al. (2016), whose findings recommended liaison with medical and psychosocial staff as an essential component for hospital teachers to be effective. Similarly, HOPE (2000) advocates that hospital teachers be full members of the multidisciplinary caring team. Findings from this research affirm that many teachers report attendance at weekly psychosocial meetings as an essential part of their role as a hospital teacher. Attendance at such meetings, where specific cases are discussed, were reported by participants as being of paramount importance when engaging with children and their parents. In agreement with findings by Äärelä et al. (2018), knowledge of the child’s background, condition, and capabilities was reported as being essential for teachers when formulating an educational plan. The home settings and experiences of the heterogeneous groupings that these teachers deal with are diverse, and having this knowledge further enhances the best approach for the hospital teacher to adopt.

One significant finding from this study, not previously referred to in the literature, was participants’ engagement with annual medical education sessions. Delivered by specialists in specific disciplines such as cystic fibrosis, cardiology, oncology/haematology, and epilepsy, among others, medical education sessions inform teachers of the features of a particular health condition and the potential side effects of treatment. Participants lauded the benefits of such medical education sessions for augmenting their knowledge of specific medical conditions and the effect that these conditions may have on a child’s propensity to access education.

A further finding not previously documented in the literature was the concept of *Teachmeet*, described as a method of sharing effective teaching methodologies and resources with neighbouring hospital school employees. There was consensus across the data set that this practice is beneficial to hospital teachers as a means of enabling them to provide a holistic curriculum to the children they encounter, encompassing more than formal curriculum learning and tailored to the special needs that may arise from illness and hospitalisation. Aligning with HOPE (2000), which hold as a principle that a variety of teaching methods and resources, differentiated for each child, be utilised in hospital education, the practice of sharing knowledge through *Teachmeet* is effective practice for acquiring knowledge of a variety of methodologies and experiences necessary to fulfil this aim.

All participants emphasised the importance of engaging with CPD as an important element of their practice. This affirms the hypothesis of the DES (2016) standard, which advocates for educators to engage in a range of CPD courses to acquire the requisite subject and pedagogical knowledge to be effective in their practice of delivering education to children. As hospital teachers deliver individualised instruction to a heterogeneous cohort of students, HOPE (2000) advocates the necessity for hospital teachers to engage with CPD to keep up-to-date with developments in education. Corroborating this hypothesis, Steinke et al. (2016) value familiarity with a variety of resources and knowledge of the curricula being delivered in base schools as intrinsic to the delivery of education to children who present to hospital school.

A further example of knowledge sharing was attendance at biennial HOPE conferences, with many participants commending these conferences for facilitating the sharing of perspectives and resources used in other hospital schools, and welcoming the opportunity to participate in such learning experiences targeted solely for hospital teachers.

### Challenges Experienced

Perspectives of hospital school teachers were also sought to identify the challenges they experience working in a hospital school setting. All teachers emphasised the emotional aspect of teaching in a children’s hospital school as a challenge (Lemke, 2004). Bereavement was identified by the majority of interviewees as the most difficult challenge, while learning that a child has transitioned to palliative care was cited by two participants as their most difficult challenge emotionally.

Time constraints were a further challenge experienced by some participants, reinforcing findings reported by Steinke et al. (2016) and echoing Lemke (2004), who noted that canvassing for students, communicating with the child and stakeholders, assessing levels, and designing educational programmes posed a significant challenge by taking up a lot of teachers’ time otherwise designed to deliver an education session to students. Further, significant demands on a hospital teacher’s time are increased as additional duties to standard teaching duties must be undertaken. These duties include reviewing the hospital admissions list, communicating with the child, his or her parents, and the medical team, rescheduling pre-planned education sessions due to medical procedures, or the status of the child’s condition. Participants also reported that on occasion they do not have time to teach all available children on their ward or are required to curtail the length of education sessions due to time restrictions.

Teaching across a variety of class levels was disclosed by Irish hospital teachers as a further challenge. Delivering effective education within a hospital setting not only requires teachers to be adept in differentiating across a variety of levels of need but also necessitates familiarity with the curricular content for a range of class levels. This requirement, combined with the reality of having to teach children who are new to the environment and require additional nurturing to settle them into their new surroundings, along with the difficulties in engaging reluctant learners, was reported as being significant demands of a teacher in a hospital school. This finding echoes those expressed by Capurso and Dennis (2017), who maintained that the hospital environment should be designed to support a sense of belonging for children where they have an active and recognised role, a concept that is challenging to implement.

## Conclusion

This qualitative research study examined the service that Irish hospital schools provide for students with medical needs to identify and document examples of the individual and collaborative practice they employ to provide for education as well as the overall challenges experienced. The findings demonstrate that hospital school teachers in Ireland engage in a variety of individual and collaborative practices to undertake their work. Notable findings detail how they identify candidates for instruction, the individual practices employed to engage children, and the process of developing educational programmes.

The findings also revealed the range of individual and collaborative practices employed to prepare for and deliver education to hospitalised students. Findings suggest that teachers in Ireland support children in hospital by providing continuity of education and endeavour to provide some normality in the atypical setting of hospital school. Specifically, teachers provide opportunities for socialisation, structure, and academic development while maintaining contact with the child’s class and class teachers through regular and structured communication. Participants expressed their belief in the importance of using high-interest individualised activities to build relationships and encourage school and learning engagement.

The research study illuminated the daily routine of planning for a child’s education, comprising a process of information gathering and recording, multidisciplinary collaboration, and engagement strategies. Findings indicated that all participants endeavour to enhance their knowledge regularly to better provide for the children they teach by engaging in medical education sessions and annual CPD.

Participating hospital teachers reported the emotional aspect of their role as the biggest challenge they experience, with the death of a child being the most commonly cited. Time constraints, teaching across a variety of class levels and needs, and a lack of recognition of the parameters within they must operate, were further challenges identified.

The findings that emerged from this study provide valuable insights into hospital education from an Irish perspective and increase the knowledge and understanding of the practices employed in hospital schools, including the fundamental challenges that accompany educating within a hospital environment. As such, the study serves to further our understanding of the hospital education system from an Irish perspective and corroborates the need for further research and acknowledgment of this essential area of education.

## Additional File

The additional file for this article can be found as follows:

10.5334/cie.25.s1 Appendix A. Interview schedule.

## Limits of the Study

The perspectives and experiences of participants represented in this qualitative study represent a small sample size and, therefore, cannot be generalised. However, the data may be used to initiate a Delphi round on a larger scale to establish agreement and to identify additional themes that could potentially emerge with a greater number of participants.

## Implications for Further Research

The support of the Professional Development Service for Teachers (PDST) and the Special Education Support Service (SESS) in providing CPD tailored to staff working in hospital school settings is beneficial; however, a comprehensive system of CPD relating to teaching students with medical needs should be established for primary and post-primary teachers across all school settings in Ireland.

This study reported the benefits of engaging in *Teachmeet* as an effective means of sharing practices and useful resources among hospital teachers. Similar collaboration between teaching staff would be of benefit to schools nationwide as a means of sharing diverse methodologies and useful practices.

A review of the criteria required by the DES inspectorate office during whole-school evaluations of hospital schools would serve to acknowledge the unique parameters within which hospital educators work. Such a review must be mindful of the unique setting and the inherent differences from the mainstream and special schools to which the initial evaluation criteria were based. Furthermore, a review of the pupil-teacher ratio of hospital schools would be beneficial in improving the provision of hospital education. Mindful of the time constraints and reality of having to canvas for students, engage in liaison with multiple stakeholders, and compete with scheduling of procedures and other hospital therapies, a ratio of 10:1 is unsustainable.
