# Supplementary material for: Continuing Education in Irish Hospital Schools: Provision for and Challenges for Teachers
Source: Contin Educ. 2021 Apr 15;2(1):42–59. doi: 10.5334/cie.25 (PMC11104316; doi:10.5334/cie.25)
Supplement: Appendix A. — Interview schedule. [file cie-2-1-25-s1.pdf]

Appendix A: Interview Schedule

1. I would like to begin by asking you how long you have been teaching in a hospital setting?

2. What was your teaching experience prior to becoming a hospital teacher?

3. Can you tell me more about how you came to be a teacher in a hospital setting?

4. Can you describe what a typical day in work is like for you?

5. Would you mind telling me about some of the children you work with?

6. What do you think are the benefits for children who receive education while they are in hospital?

7. Do you think there are any drawbacks for children who do not participate in school while in hospital?

8. Can you talk me through the process of how you identify which children are candidates for receiving school while in hospital?

9. How would you go about gathering information about a new student?

10. How do you introduce school to a new student?

11. Can you talk me through the process of how you engage with a child?

12. In your experience, do different medical conditions affect a child's ability to learn?

13. If so, how do you become aware of how a particular health condition may impact a child's ability to learn?

14. What type of planning takes place for the child's education?

15. Who do you engage with to create a plan for a child?

16. Do you find there to be a difference between how you plan for a child who may be in for a short stay versus a long-term/recurring patient? If so can you tell me more about how you engage in such planning?

17. Hospital teachers teach across many class levels and needs. In your opinion what are challenges of this?

18. How do you keep up to date with the range of educational programmes, developments and new initiatives in place around the country?

19. Do you get the opportunity to discuss teaching strategies with other hospital teachers? If so where and when do these professional conversations take place?

20. Can you give me some examples of extra training/CPD you have engaged in to enhance your teaching of children with medical needs?

21. Aside from your teaching qualification, have you engaged in any other professional development that has been beneficial to you when working in this setting?

22. In your opinion, what challenges do children face while in hospital school?

23. The children are away from their peers during hospitalisations, do you see this as having any effect on their participation in school while they are in hospital? If so, how do you address this?

24. Do you find there are some reluctant learners?

25. How do you engage reluctant learners?

26. The hospital school setting differs greatly from mainstream and special school settings, are there any particular challenges that come to mind for you?

27. In your experience, what do you most enjoy about your job as a teacher in a hospital setting?

28. What do you feel is the most difficult aspect of your job?

29. Are there supports in place to help you deal with this challenge?

30. Are there other challenges you face here that you had not encountered before teaching in a hospital setting?

31. Would you like to ask me any questions before we conclude this interview or indeed add anything you may have not mentioned previously?
